# miR-124-3p functions as a tumor suppressor in breast cancer by targeting CBL

**DOI:** 10.1186/s12885-016-2862-4

**Published:** 2016-11-15

**Authors:** Yanbo Wang, Luxiao Chen, Zhenyu Wu, Minghai Wang, Fangfang Jin, Nan Wang, Xiuting Hu, Zhengya Liu, Chen-Yu Zhang, Ke Zen, Jiangning Chen, Hongwei Liang, Yujing Zhang, Xi Chen

**Affiliations:** 1 State Key Laboratory of Pharmaceutical Biotechnology, Jiangsu Engineering Research Center for MicroRNA Biology and Biotechnology, NJU Advanced Institute for Life Sciences (NAILS), School of Life Sciences, Nanjing University, 163 Xianlin Road, Nanjing, Jiangsu 210046 China; 2 Department of General Surgery, The First Affiliated Yijishan Hospital with Wannan Medical College, 2 West Zheshan Road, Wuhu, Anhui 241001 China

**Keywords:** miR-124-3p, Breast cancer, Proliferation, Invasion, CBL

## Abstract

**Background:**

The origin and development of breast cancer remain complex and obscure. Recently, microRNA (miRNA) has been identified as an important regulator of the initiation and progression of breast cancer, and some studies have shown the essential role of miR-124-3p as a tumor suppressor in breast tumorigenesis. However, the detailed role of miR-124-3p in breast cancer remains poorly understood.

**Methods:**

Quantitative RT-PCR and western blotting assays were used to measure miR-124-3p and CBL expression levels in breast cancer tissues, respectively. Luciferase reporter assay was employed to validate the direct targeting of CBL by miR-124-3p. Cell proliferation and invasion assays were performed to analyze the biological functions of miR-124-3p and CBL in breast cancer cells.

**Results:**

In the present study, we found that miR-124-3p was consistently downregulated in breast cancer tissues. Moreover, we showed that miR-124-3p significantly suppressed the proliferation and invasion of breast cancer cells. In addition, we investigated the molecular mechanism through which miR-124-3p contributes to breast cancer tumorigenesis and identified CBL (Cbl proto-oncogene, E3 ubiquitin protein ligase) as a direct target gene of miR-124-3p. Moreover, we found that ectopic expression of CBL can attenuate the inhibitory effect of miR-124-3p on cell proliferation and invasion in breast cancer cells.

**Conclusions:**

This study identified a new regulatory axis in which miR-124-3p and CBL regulate the proliferation and invasion of breast cancer cells.

**Electronic supplementary material:**

The online version of this article (doi:10.1186/s12885-016-2862-4) contains supplementary material, which is available to authorized users.

## Background

Breast cancer is the leading cause of death in women and is the most common type of cancer. Although advances in the diagnosis and treatment of breast cancer have greatly reduced its incidence and mortality, there are still 500,000 breast cancer deaths per year worldwide [1, 2]. An improved understanding of the molecular mechanisms of this disease is needed for developing new treatments.

MicroRNAs (miRNAs) are non-coding RNAs that are small size and single stranded and regulate most of biological processes, including cell proliferation, differentiation, migration and apoptosis. MiRNAs cause post-transcriptional silencing of target mRNAs by binding to their 3’-untranslated regions (3’-UTRs) through complementary base pairing [3]. During the initial and subsequent developmental stages of human cancers, miRNAs can function either as oncogenes or tumor suppressors [4–6]. For example, miR-124-3p, a brain-enriched miRNA involved in the regulation of gastrulation and neural development [7, 8], has recently been reported to function as a tumor suppressor through targeting some important genes, such as RAC1, the androgen receptor, SPHK1, ROCK2 and EZH2 [9]. Although these studies have shown the potential role of miR-124-3p as a tumor-suppressive miRNA during tumorigenesis [10, 11], the detailed function of miR-124-3p in the initiation and progression of breast cancer remains poorly understood.

In this study, we measured the expression levels of miR-124-3p in 10 pairs of breast cancer and matched adjacent noncancerous tissue samples and found that miR-124-3p levels were downregulated in breast cancer tissues. Using bioinformatics algorithms, we predicted CBL (Cbl proto-oncogene, E3 ubiquitin protein ligase) as a target gene of miR-124-3p. Furthermore, we showed that miR-124-3p could suppress CBL expression and negatively regulate the proliferation and invasion of breast cancer cells.

## Methods

### Cells and human tissues

The human breast cancer cell lines MCF-7 and MDA-MB-231 were obtained from the Shanghai Institute of Biochemistry and Cell Biology, Chinese Academy of Sciences (Shanghai, China). MCF-7 and MDA-MB-231 cells were cultured in DMEM and L15, respectively; both media were supplemented with 10 % fetal bovine serum (FBS, GIBCO, CA, USA) and were placed in incubator at 37 °C in a humidified 5 % CO_2_ atmosphere. Breast cancer and normal adjacent tissues are provided by the The First Affiliated Yijishan Hospital with Wannan Medical College (Wuhu, China) from patients during surgery. All protocols concerning the use of patient samples in this study were approved by the Medical Ethics Committee of the The First Affiliated Yijishan Hospital with Wannan Medical College (Wuhu, China) All samples collection is done following guidelines of Institutional Review Board–approved protocol after a written agreement approval by the patients. Liquid nitrogen is used to freeze samples soon after their collection from surgery and later on stored at -80 °C. The clinical features of the patients are listed in Additional file 1: Table S1.

### RNA isolation and quantitative RT-PCR

TRIzol Reagent (Invitrogen, Carlsbad, CA) is used to extract total RNA according to manufacturer’s guidelines. Stem-loop quantitative RT-PCR (qRT-PCR) assays using TaqMan miRNA probes (Applied Biosystems) were performed to quantify the levels of mature miRNAs on Applied Biosystems 7300 Sequence Detection System (Applied Biosystems). The reactions were incubated in a 96-well optical plate at 95 °C for 10 min, followed by 40 cycles at 95 °C for 15 s and 60 °C for 1 min. The relative levels of the miRNAs in cells and tissues were normalized to U6. By using the 2^-△△CT^ equation, miRNA amount was calculated relative to the internal control U6, where △△C_T_ = (C_T miRNA_ - C_T U6_)_tumor_ - (C_T miRNA_ - C_T U6_)_control_.

To quantify CBL mRNA, 1 μg of total RNA was reverse-transcribed to cDNA using oligo dT and AMV reverse transcriptase (TaKaRa, Dalian, China) and incubated at 16 °C for 30 min, followed by 42 °C for 30 min and 85 °C for 5 min. Real-time PCR was performed using SYBR Green Dye (Invitrogen) and CBL and GAPDH primers. Primers sequences were: CBL (sense): 5’-TGACATCTTTACCCGACTC-3’; CBL (antisense): 5’-CATACCCAATAGCCCAC-3’; GAPDH (sense): 5’-GATATTGTTGCCATCAATGAC-3’; and GAPDH (antisense): 5’-TTGATTTTGGAGGGATCTCG-3’. The relative amount of CBL mRNA was normalized to GAPDH.

### miRNA overexpression or knockdown

In order to achieve miR-124-3p overexpression, cells were transfected with miR-124-3p mimic (a mimicking precursor of miR-124-3p that is double-stranded synthetic RNA oligonucleotide) as previously described [12]. Knockdown of miR-124-3p was achieved by transfecting cells with miR-124-3p antisense (a chemically modified antisense oligonucleotide designed to target mature miR-124-3p) as previously described [12]. Synthetic miR-124-3p mimic (pre-miR-124-3p), antisense (anti-miR-124-3p) and scrambled negative control RNAs (pre-miR-control and anti-miR-control) were bought from GenePharma (Shanghai, China). MCF-7 and MDA-MB-231 were transfected with Lipofectamine 2000 (Invitrogen) in 6-well plates when cells were approximately 70 % confluent. Old medium was replaced with new DMEM or L15 supplemented with 2 % FBS after 6 h.

A mammalian expression plasmid encoding human CBL open reading frame (ORF) was obtained from GeneCopoeia (pReceiver-M02-CBL, Germantown, MD, USA). For negative control, an empty plasmid was used. The siRNA (sequence: 5’-CCUAGUCUCCUCUAUCGCUTT-3’) targeting human CBL was designed and synthesized by GenePharma. A scrambled siRNA (GenePharma) was also used as a negative control. MCF-7 cells were transfected with overexpression plasmid or siRNA by using Lipofectamine 2000 (Invitrogen) following manufacturer’s instructions. After 24 or 48 h, total RNA or protein were extracted.

### Western blotting

The protein levels were analyzed by western blot using the appropriate antibodies. Anti-GAPDH antibody was applied to the same blots to normalize protein levels. Detail of antibodies and corresponding sources from where these were purchased are as follows: anti-c-CBL (610442; BD Biosciences, USA) and anti-GAPDH (sc-365062; Santa Cruz Biotechnology, Santa Cruz, CA, USA). ImageJ software was used for protein bands analysis.

### Luciferase reporter assay

Luciferase reporter assay was done as previously described to predict the direct binding of miR-124-3p to the target gene CBL [13]. PGL3 plasmid encoding a luciferase report gene was purchased from Ambion. The recombinant plasmid PGL3-CBL-3’-UTR was constructed by inserting the sequence of CBL 3’-UTR into the PGL3 plasmid. DNA sequencing was performed to confirm successful insertion. The mutant 3’-UTR sequence of CBL was directly synthesized and inserted into the equivalent reporter plasmid by GenePharma (all three binding sites were mutated). For luciferase reporter assay, HEK293T cells were transfected using Lipofectamine 2000 (Invitrogen) by seeding HEK293T cells in 24-well plates and then each well of the plate was transfected with 0.1 μg of a β-galactosidase (β-gal) expression plasmid (Ambion), 0.1 μg of firefly luciferase reporter plasmid and 100 pmol of pre-miR-124-3p. Scrambled RNA was used as a negative control and β-gal plasmid as a transfection control. The cells were assayed using a luciferase assay kit 24 h after transfection (Promega, Madison, WI, USA).

### Cell proliferation assay

For each well in 96-well plates, 5 × 10^3^ MCF-7 cells were seeded and transfected 24 h later. After transfection, 10 μl WST-8 solution from the Cell Counting Kit-8 (CCK8, Beyotime, China) was added into each well. Absorbance of each well was measured after 2 h incubation by reading plates at 450 nm at time 12, 24, 36 and 48 h. The relative cell number was calculated as the ratio of absorbance at 24, 36 and 48 h to12 h.

### Cell invasion assay

Transwell invasion assay was performed using Transwell Boyden Chambers (6.5-mm, Costar, USA) with inserts that have membrane with pore size of 8-μm and a slim Matrigel layer. About 12 h after transfection, cells were harvested, suspended in DMEM culture medium that did not contain FBS and then loaded to upper chamber (2 × 10^4^ cells/well). Then 500 μl DMEM medium that contained 10 % FBS was added to the lower compartment at the same time and the plates were placed for incubation for 7 h in 5 % CO_2_-humidified atmosphere. After 7 h incubation, lower surface of the filter membrane that contained migrant cells was collected and fixed with 4 % paraformaldehyde at room temperature for 25 min. Membrane washing is done by distilled water for three times and the membrane is then stained with 0.1 % crystal violet in methanol at room temperature for 15 min. Non-migrant cells that remained on the upper surface of the filter membrane were collected by gentle scrapping using a cotton swab. Migrant cells in lower surface were analyzed by photomicroscope (BX51 Olympus, Japan), and the cells were counted blindly (five fields per chamber).

### Statistical analysis

All data of western blot images presented here is selected from minimum three separate experiments. Luciferase reporter assay, qRT-PCR assay, cell proliferation and invasion assay were conducted three times and with many repetitions. The results presented here is the means ± SE of minimum three separate experiments. The differences were analyzed by Student’s *t*-test and considered statistically significant at *P* < 0.05.

## Results

### Expression patterns of miR-124-3p in breast cancer tissues

**Fig. 1:**
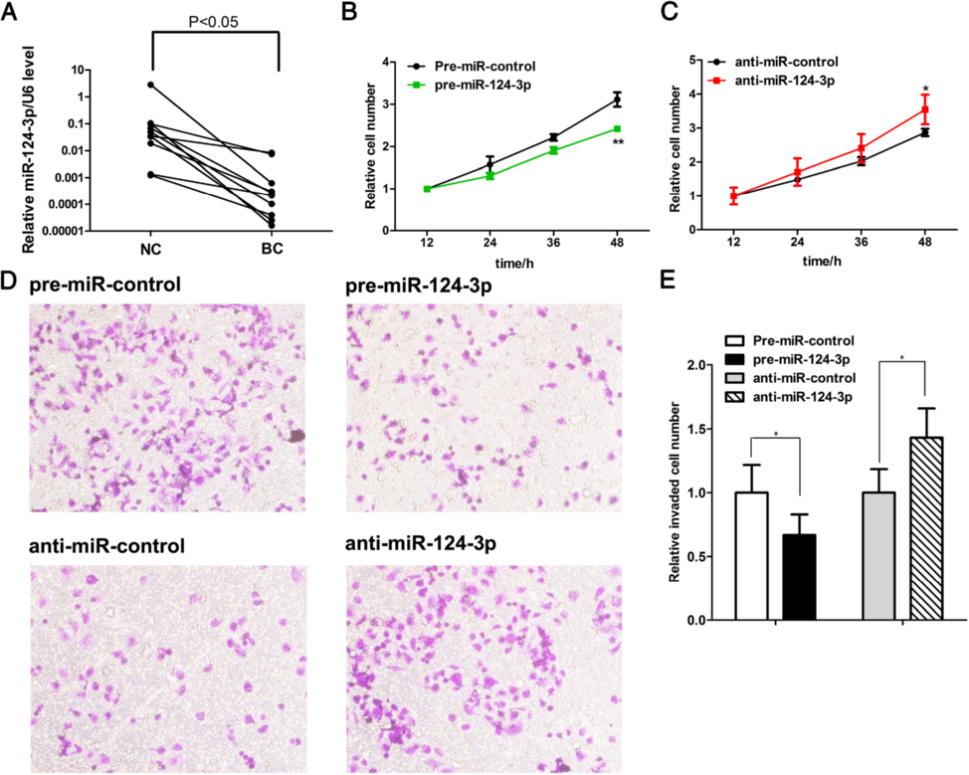
miR-124-3p functions as a tumor suppressor in breast cancer. **a** qRT-PCR analysis of the expression levels of miR-124-3p in 10 pairs of breast cancer tissue (BC) and noncancerous tissue (NC) samples. **b** and **c** CCK8 analysis of the proliferation rate in MCF-7 cells treated with control mimic or miR-124-3p mimic (**b**), or with control antisense or miR-124-3p antisense (**c**). **d** and **e)** Transwell analysis of invaded MCF-7 cells treated with control mimic, miR-124-3p mimic, control antisense or miR-124-3p antisense. **d** representative image; **e** quantitative analysis. *P < 0.05; **P < 0.01

To investigate the roles of miR-124-3p in human breast cancer, we first determined the expression pattern of miR-124-3p in breast cancer tissues. By measuring the miR-124-3p levels in 10 pairs of breast cancer tissues and normal adjacent tissues, we found that miR-124-3p levels were consistently downregulated in breast cancer tissues (Fig. 1a).

### miR-124-3p functions as a tumor suppressor in breast cancer cells

Next, we investigated the biological effects of miR-124-3p on breast cancer cells. First, we overexpressed miR-124-3p in a human breast cancer cell line (MCF-7) and used CCK8 assay to examine the effect of miR-124-3p on cell proliferation. We found that the number of proliferating cells was significantly reduced after transfection of MCF7 cells with miR-124-3p mimic but increased after transfection with miR-124-3p antisense (Fig. 1b and c). Additionally, we checked the effects of miR-124-3p on cell invasion using the Transwell assay. The invasion rate was significantly decreased after transfection of MCF7 cells with miR-124-3p mimic but increased after transfection with miR-124-3p antisense (Fig. 1d and e). These data indicate that miR-124-3p might inhibit the proliferation and invasion of breast cancer cells and act as a tumor-suppressive miRNA during breast tumorigenesis.

### Prediction of CBL as a target of miR-124

**Fig. 2:**
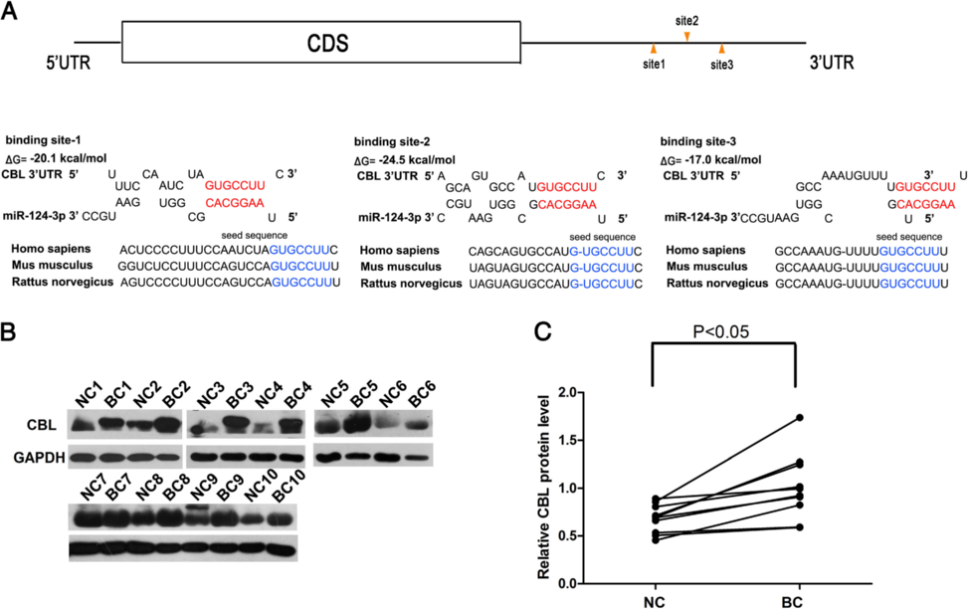
Prediction of CBL as a target of miR-124-3p. **a** Diagram illustrating the hypothetical duplexes formed by interactions between the binding sites in the CBL 3’-UTR (top) and miR-124-3p (bottom). The predicted free energy value of each hybrid is indicated. The seed recognition sites are denoted; all nucleotides in these regions are highly conserved across species, including human, mouse and rat. **b** and **c** Western blot analysis of the expression levels of CBL protein in 10 pairs of BC and NC samples. **b** representative image; **c** quantitative analysis

To explore the mechanism through which miR-124-3p influences breast cancer cell proliferation and invasion, a list of predicted targets of miR-124-3p was compiled using the bioinformatics algorithms TargetScan [14], miRanda [15] and PicTar [16]. Among the candidates, CBL, an oncogene that is frequently observed to be upregulated in breast cancer [17], was selected and further investigated. As shown in Fig. 2a, there were three predicted targeting sites of miR-124-3p within the 3’-UTR of CBL. The minimum free energy values of the three hybridizations were -17.0, -20.1 and -24.5 kcal/mol, and these hybridizations had perfect base-pairing between the seed sequences (the 2^nd^ to 8^th^ bases of the mature miRNA) and the cognate targets.

We next examined if miR-124-3p had expression patterns that are opposite to CBL in breast cancer. By measuring CBL protein levels in the same 10 pairs of breast cancer tissues and normal adjacent tissues, we found that CBL was consistently upregulated in the breast cancer tissues (Fig. 2b and c). Thus, CBL was selected as a target of miR-124-3p, based on both computational predictions and their inverse correlation in breast cancer tissues.

### Validation of the direct targeting of CBL by miR-124-3p

**Fig. 3:**
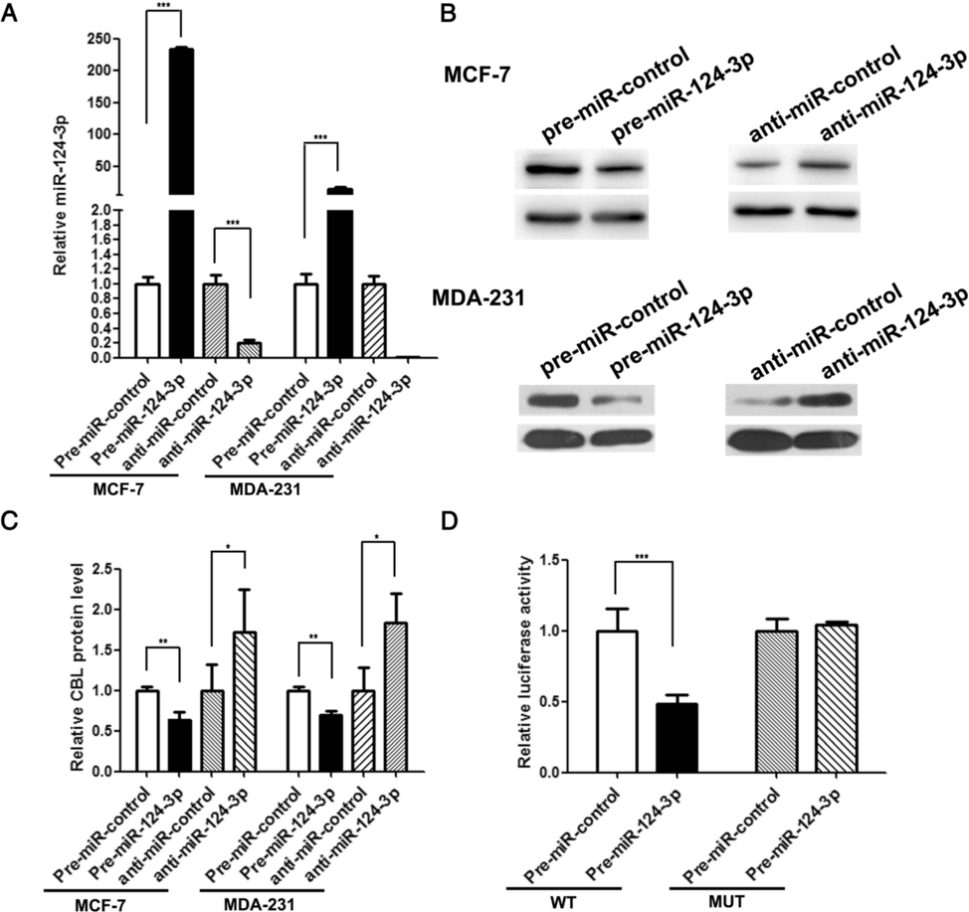
CBL is a direct target of miR-124-3p. **a** qRT-PCR analysis of the expression levels of miR-124-3p in MCF-7 and MDA-MB-231 cells treated with control mimic, miR-124-3p mimic, control antisense or miR-124-3p antisense. **b** and **c** Western blot analysis of the expression levels of CBL protein in MCF-7 and MDA-MB-231 cells treated with control mimic, miR-124-3p mimic, control antisense or miR-124-3p antisense. **b** representative image; **c** quantitative analysis. **d** Firefly luciferase reporters containing wild-type (WT) or mutant (MUT) miR-124-3p binding sites in the CBL 3’-UTR were co-transfected into HEK293T cells together with control mimic or miR-124-3p mimic. The cells were assayed using a luciferase assay kit 24 h post-transfection. *P < 0.05; **P < 0.01; ***P < 0.001

To further validate the correlation between miR-124-3p and CBL, we overexpressed or knocked down miR-124-3p in MCF-7 and MDA-MB-231 breast cancer cell lines and assessed the protein levels of CBL. As anticipated, the cellular levels of miR-124-3p were dramatically increased in MCF-7 and MDA-MB-231 cells after transfection with miR-124-3p mimic and dropped significantly after treatment with miR-124-3p antisense (Fig. 3a). Consequently, CBL protein levels were decreased in MCF-7 and MBA-MD-231 cells by the introduction of miR-124-3p, while the antisense of miR-124-3p significantly increased the CBL protein levels in these two breast cancer cell lines (Fig. 3b and c).

Subsequently, a luciferase reporter assay was performed to confirm that miR-124-3p directly targets the predicted binding sites in the CBL 3’-UTR and negatively regulates CBL expression. The CBL 3’-UTR sequence was inserted downstream of the firefly luciferase gene in a reporter plasmid. The recombination plasmid was transfected along with miR-124-3p mimic into HEK293T cells. As expected, the luciferase activity was markedly reduced in cells co-transfected with luciferase reporter plasmid and miR-124-3p mimic (Fig. 3d). We then introduced point mutations into the predicted miR-124-3p binding sites in the CBL 3’-UTR to eliminate the potential interaction between miR-124-3p and CBL. As a result, overexpression of miR-124-3p had no effect on the mutated luciferase reporter activity (Fig. 3d), suggesting that the binding sites strongly contribute to the interaction between miR-124-3p and CBL. In summary, miR-124-3p directly binds to the CBL 3’-UTR and suppresses CBL translation.

### miR-124-3p suppresses tumor proliferation and invasion through targeting CBL

**Fig. 4:**
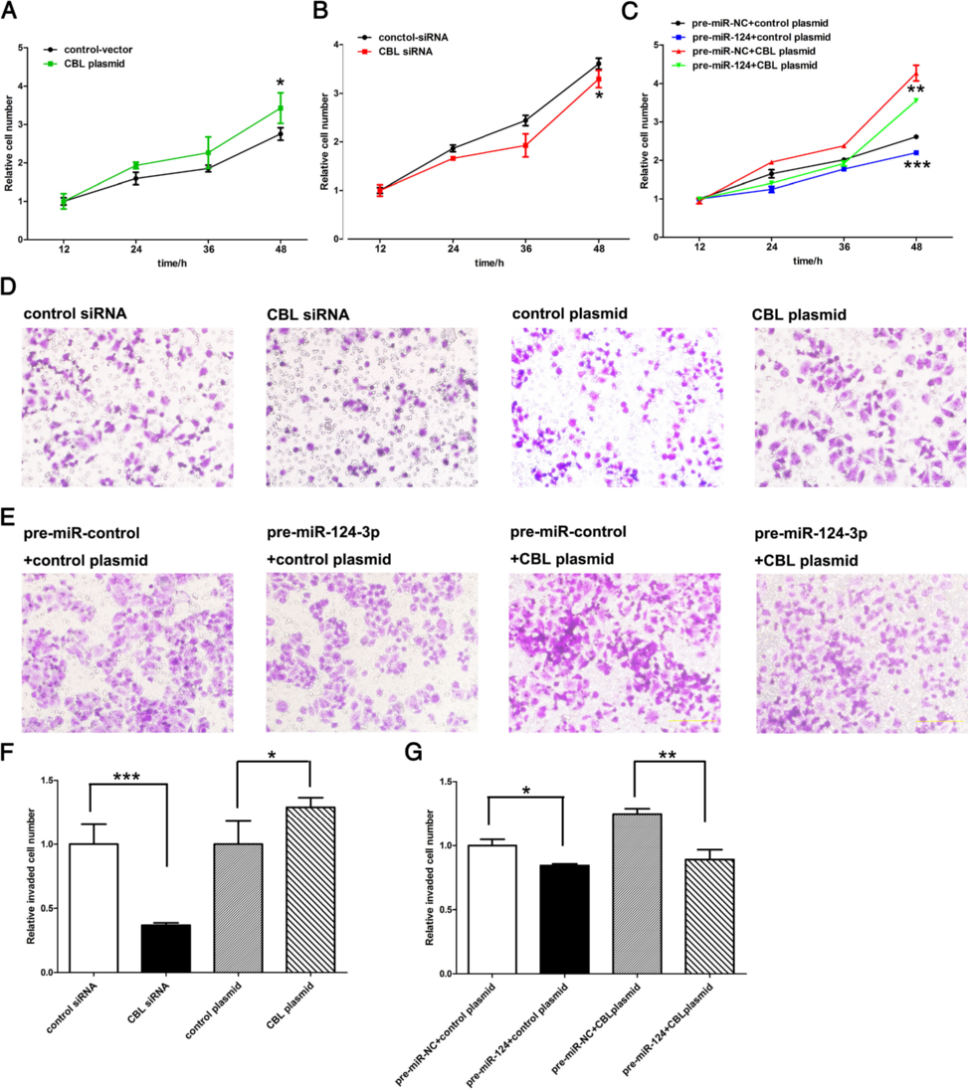
Effects of miR-124-3p and CBL on the proliferation and invasion of MCF-7 cells. **a**–**c** CCK8 analysis of the proliferation rate of MCF-7 cells treated with control siRNA or CBL siRNA (**a**), with control plasmid or CBL plasmid (**b**), or with one of the combinations: control mimic plus control plasmid, miR-124-3p mimic plus control plasmid, control mimic plus CBL plasmid, or miR-124-3p mimic plus CBL plasmid (**c**). **d**–**g** Transwell analysis of invaded MCF-7 cells treated with control siRNA or CBL siRNA (**d**), with control plasmid or CBL plasmid (**d**), or with one of the combinations: control mimic plus control plasmid, miR-124-3p mimic plus control plasmid, control mimic plus CBL plasmid, or miR-124-3p mimic plus CBL plasmid (**e**). **d** and **e** representative images; **f** and **g** quantitative analysis. *P < 0.05; **P < 0.01; ***P < 0.001

We next analyzed the biological consequences of the repression of CBL by miR-124-3p. Because CBL is well known to promote tumor proliferation and invasion [18, 19], we examined if miR-124-3p would modulate CBL to influence the proliferation and invasion of breast cancer cells. We designed a siRNA sequence targeting CBL ORF to knockdown CBL and constructed a plasmid expressing the full-length CBL ORF to overexpress CBL without the miR-124-responsive 3’-UTR. Efficient overexpression or knockdown of CBL was observed in MCF-7 cells after transfection with CBL overexpression plasmid and CBL siRNA, respectively (Additional file 2: Figure S1). Transfection of CBL siRNA markedly reduced the number of proliferating MCF-7 cells, whereas transfection of the CBL overexpression plasmid promoted cell proliferation (Fig. 4a and b). Thus, the inhibition of cell proliferation by CBL knockdown is equivalent to miR-124-3p overexpression. Additionally, cells transfected with miR-124-3p mimic and the CBL overexpression plasmid showed higher proliferation rates compared to cells transfected with miR-124-3p mimic alone (Fig. 4c), suggesting that miR-124-3p-resistant CBL could sufficiently rescue the suppression of CBL by miR-124-3p and attenuate the anti-proliferative effect of miR-124-3p on breast cancer cells.

We also evaluated the effects of miR-124-3p-mediated suppression of CBL expression on the invasion of breast cancer cells using the Transwell invasion assay. Silencing of CBL expression using specific siRNA significantly decreased the number of MCF-7 cells that invaded through the Transwell membrane, while overexpression of CBL using specific plasmid markedly promoted cell invasion (Fig. 4d and f). Thus, the inhibition of cell invasion by CBL knockdown is equivalent to miR-124-3p overexpression. Additionally, when MCF-7 cells were simultaneously transfected with the miR-124-3p mimic and the CBL overexpression plasmid, CBL dramatically attenuated the inhibitory effect of miR-124-3p on cell invasion (Fig. 4e and g). In summary, miR-124-3p might suppress the proliferation and invasion ability of breast cancer cells by targeting CBL.

## Discussion

Breast cancer cause highest number of deaths globally and is the most common type of cancer among women [20, 21]. The development of new treatments is halted mainly because of drug resistance and less knowledge about tumor cell signaling pathways. Over the past decade, a class of small, non-coding, single-stranded RNAs known as miRNAs has emerged as major regulators of the initiation and progression of human cancers, including breast cancer [22, 23]. Importantly, dysregulated and dysfunctional miRNAs play a causal role in cancer etiology because miRNAs can affect the translation and stability of targeted oncogenes and tumor suppressors, which eventually influences cellular physiology [4–6]. In the present study, we detected significant reduction of miR-124-3p in breast cancer tissues. As one of the most enriched miRNAs in the brain of mammals [24], miR-124-3p is involved in both brain development and neuronal function [25, 26]. Recently, miR-124-3p has been identified as a tumor suppressor in some cancers, including hepatocellular carcinoma [27], cervical cancer [28] and gastric cancer [29]. However, the function of miR-124-3p in breast cancer is largely unknown. In the present study, we found that miR-124-3p can suppress the proliferation and invasion of breast cancer cells, suggesting that miR-124-3p may play a critical role in the negative regulation of growth and metastasis of breast cancer. Accumulating evidence suggests that proliferation and invasion of cells are very important in carcinogenesis and its progression [30]. The results indicate that miR-124-3p may serve as an ideal therapeutic target for breast cancer. Additional work is needed to characterize the feasibility of targeting miR-124-3p in cancer therapy and develop simplified and cost-effective methods.

The CBL families are highly conserved ubiquitin ligases. Thus far, three mammalian homologs have been defined — CBL (also known as c-CBL), CBL-b and CBL-c — which vary from one another on the basis of difference in length of C termini and their property to work as adaptors [31]. Among the three homologs of the family, CBL is mainly known as a ubiquitin E3 ligase that is responsible for signal transduction in different cell types against various types of stimuli [32, 33]. It is thought that the predominant function of CBL is causing ubiquitination of active RTKs thereby resulting in negative regulation of their signaling and directing them towards lysosomes to degrade [34]. From this point of view, CBL may act mainly as a tumor suppressor in the pathogenesis of human cancers. For example, some studies revealed a role of CBL in restricting tumor cell proliferation and invasion [35, 36]. Conversely, CBL is regarded as a proto-oncogene with numerous mutations and important roles in some cancers, including myeloid neoplasms [37], colorectal cancer [38] and glioma [19]. Thus, if CBL acts as a tumor suppressor or an oncogene is dependent on the cell and tumor types. Under different circumstances, CBL may exert opposite functions. In breast cancer, the expression profile of CBL has not been systematically investigated and the precise function of this gene remains unclear. Overall, the findings of this study demonstrated that CBL is overexpressed in human breast cancer tissues and that the aberrant expression of CBL is responsible for the malignant behaviors of breast cancer cells. Consistently, it was reported that CBL blocks the tumor suppressor activity of TGF-β and enhances breast tumor formation [17]. Furthermore, the molecular mechanism accounting for the aberrant upregulation of CBL in breast cancer was investigated. Mechanistic studies revealed that miR-124-3p directly binds the CBL 3’-UTR and inhibit CBL expression, and that CBL overexpression sufficiently attenuates the inhibitory effects of miR-124-3p on breast cancer cell proliferation and invasion. Therefore, the modulation of CBL by miR-124-3p may explain why the downregulation of miR-124-3p can promote the development of breast cancer.

## Conclusion

Overall, this study demonstrates that miR-124-3p possesses tumor suppressor activity by negatively regulating CBL expression in breast cancer. The findings may provide insight into the molecular mechanism of breast cancer and open new avenues for cancer therapy.

## Additional files

Additional file 1: Table S1. Patients’ Characteristics. (DOCX 17 kb)
Additional file 2: Figure S1. Downregulation of CBL by siRNA and upregulation of CBL by an overexpression plasmid in MCF-7 cells. (A-C) Western blotting analysis of CBL protein levels in MCF-7 cells treated with control siRNA, CBL siRNA, control plasmid or CBL plasmid (A and B: representative image; C: quantitative analysis). **P* < 0.05; ***P* < 0.01. (JPG 337 kb)

## Abbreviations

CBL: Cbl proto-oncogene, E3 ubiquitin protein ligase
miRNA: microRNA
