# Supplementary material for: miR-124-3p functions as a tumor suppressor in breast cancer by targeting CBL
Source: BMC Cancer. 2016 Nov 15;16:826. doi: 10.1186/s12885-016-2862-4 (PMC5109743; doi:10.1186/s12885-016-2862-4)
Supplement: Additional file 1: Table S1. — Patients’ Characteristics. (DOCX 17 kb) [file 12885_2016_2862_MOESM1_ESM.docx]

**miR-124-3p functions as a tumor suppressor in breast cancer by targeting CBL**

Yanbo Wang^1,*^, Luxiao Chen^1,*^, Zhenyu Wu^1,*^, Minghai Wang^2,*^, Fangfang Jin^1^, Nan Wang^1^, Xiuting Hu^1^, Zhengya Liu^1^, Chen-Yu Zhang^1^, Ke Zen^1^, Jiangning Chen^1^, Hongwei Liang^1,#^, Yujing Zhang^1, #^, Xi Chen^1,#^

**Additional Tables**

**Additional file 1: Table S1.** Patients’ Characteristics.

| Patients’ characteristics | | | | |  | |
| --- | --- | --- | --- | --- | --- | --- |
| Case No. | Clinical History | Age Ranges (years) | TNM Stage | Hormone Receptor | |  |
| BC #1 | IDC | 40~50 | II | ER-, PR-, HER2/neu+ | |  |
| BC #2 | IDC | 50~60 | II | ER/PR+, HER2/neu+ | |  |
| BC #3 | IDC | 40~50 | II | ER/PR+, HER2/neu+ | |  |
| BC #4 | IDC | 40~50 | II | ER/PR+, HER2/neu+ | |  |
| BC #5 | IDC | 40~50 | II | ER/PR+, HER2/neu+ | |  |
| BC #6 | IDC | 30~40 | III | ER/PR+, HER2/neu- | |  |
| BC #7 | IDC | 60~70 | III | ER/PR+, HER2/neu- | |  |
| BC #8 | IDC | 60~70 | III | ER+PR-, HER2/neu- | |  |
| BC #9 | IDC | 40~50 | III | ER/PR+, HER2/neu- | |  |
| BC #10 | IDC | 40~50 | III | ER/PR-, HER2/neu- | |  |

**Supplementary Figures**

**Supplementary Figure 1. Downregulation of CBL by siRNA and upregulation of CBL by an overexpression plasmid in MCF-7 cells. (A-C)** Western blotting analysis of CBL protein levels in MCF-7 cells treated with control siRNA, CBL siRNA, control plasmid or CBL plasmid (A and B: representative image; C: quantitative analysis). * P < 0.05; ** P < 0.01.
