# Supplementary figures and images for: miR-124-3p functions as a tumor suppressor in breast cancer by targeting CBL
Source: BMC Cancer. 2016 Nov 15;16:826. doi: 10.1186/s12885-016-2862-4 (PMC5109743; doi:10.1186/s12885-016-2862-4)

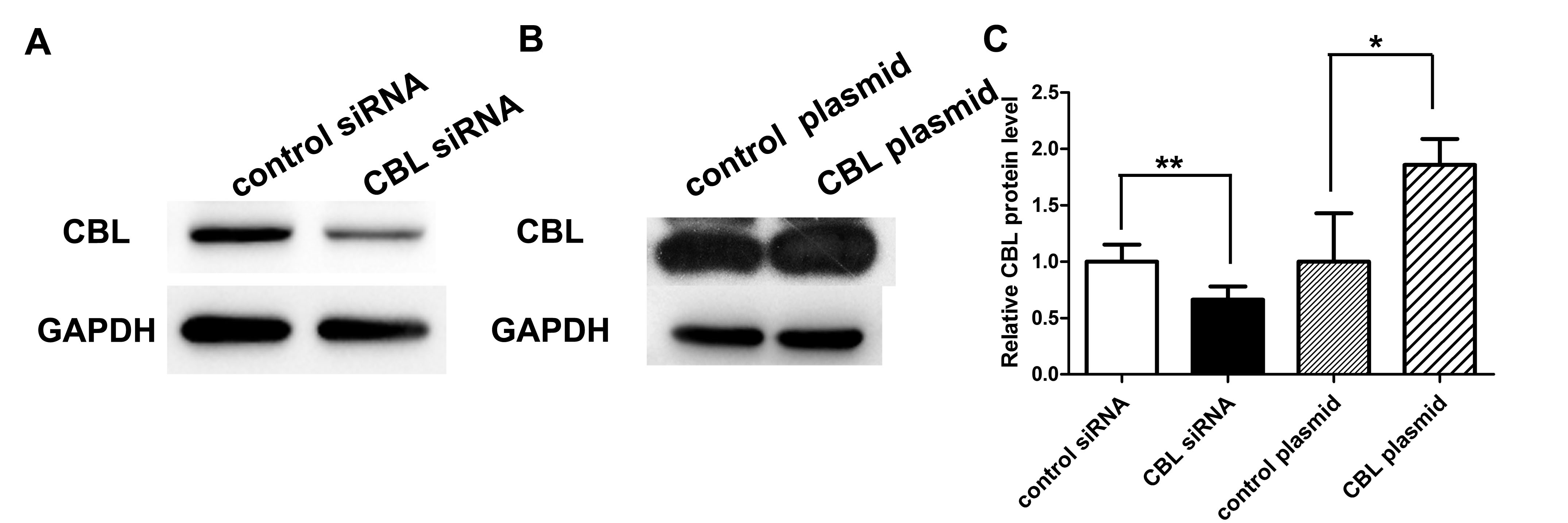

Supplement: Additional file 2: Figure S1. — Downregulation of CBL by siRNA and upregulation of CBL by an overexpression plasmid in MCF-7 cells. (A-C) Western blotting analysis of CBL protein levels in MCF-7 cells treated with control siRNA, CBL siRNA, control plasmid or CBL plasmid (A and B: representative image; C: quantitative analysis). *P < 0.05; **P < 0.01. (JPG 337 kb) [file 12885_2016_2862_MOESM2_ESM.jpg]
